# The impact of inconsistent human annotations on AI driven clinical decision making

**DOI:** 10.1038/s41746-023-00773-3

**Published:** 2023-02-21

**Authors:** Aneeta Sylolypavan, Derek Sleeman, Honghan Wu, Malcolm Sim

**Affiliations:** 1 Institute of Health Informatics, University College London, London, United Kingdom; 2 School of Natural and Computing Sciences, University of Aberdeen, Aberdeen, Scotland UK; 3 Alan Turing Institute, London, United Kingdom; 4 School of Medicine, Nursing and Dentistry, University of Glasgow, Aberdeen, Scotland UK

**Keywords:** Research data, Medical research

## Abstract

In supervised learning model development, domain experts are often used to provide the class labels (annotations). Annotation inconsistencies commonly occur when even highly experienced clinical experts annotate the same phenomenon (e.g., medical image, diagnostics, or prognostic status), due to inherent expert bias, judgments, and slips, among other factors. While their existence is relatively well-known, the implications of such inconsistencies are largely understudied in real-world settings, when supervised learning is applied on such ‘noisy’ labelled data. To shed light on these issues, we conducted extensive experiments and analyses on three real-world Intensive Care Unit (ICU) datasets. Specifically, individual models were built from a common dataset, annotated independently by 11 Glasgow Queen Elizabeth University Hospital ICU consultants, and model performance estimates were compared through internal validation (Fleiss’ *κ* = *0*.383 i.e., fair agreement). Further, broad external validation (on both static and time series datasets) of these 11 classifiers was carried out on a HiRID external dataset, where the models’ classifications were found to have low pairwise agreements (average Cohen’s *κ* = 0.255 i.e., minimal agreement). Moreover, they tend to disagree more on making discharge decisions (Fleiss’ *κ* = *0.174*) than predicting mortality (Fleiss’ *κ* = *0.267*). Given these inconsistencies, further analyses were conducted to evaluate the current best practices in obtaining gold-standard models and determining consensus. The results suggest that: (a) there may not always be a “super expert” in acute clinical settings (using internal and external validation model performances as a proxy); and (b) standard consensus seeking (such as majority vote) consistently leads to suboptimal models. Further analysis, however, suggests that assessing annotation learnability and using only ‘learnable’ annotated datasets for determining consensus achieves optimal models in most cases.

## Introduction

Classical supervised machine learning assumes the labels of training examples are all correct, ignoring the presence of class noise and inaccuracies^1^. In healthcare, this assumption may not hold even when highly experienced clinicians provide these labels, due to the degree of noise, observer subjectivity and bias involved. If neglected in the training of a Machine Learning Decision-Support-System (ML-DSS), annotation inconsistencies may result in an arbitrarily partial version of the ground truth, and to subsequent unpredictable clinical consequences, including erroneous classifications^2–4^.

Ideally, class labels are obtained via a knowledge acquisition process, involving choosing the appropriate “gold-standard” to base these ground truth class labels on, to build a Knowledge-Based System (KBS). Within the healthcare and biomedical setting, clinical domain experts are often used to provide these labels^5^. However, in many clinical areas, these ground truths are hard to find and define, due to the pathophysiological, diagnostic and prognostic uncertainties inherent to medicine^2,6^.

Cognitive Psychology has shown experimentally that humans (& hence experts) make “slips”, for example, due to cognitive overload and due to biases. On the other hand, the field of expert systems and KBS has assumed that for (most) disciplines “slip-free” highly skilled experts exist, and the key task is how such experts can be objectively or subjectively identified. However, increasing evidence from the literature shows, on common sets of (e.g., classification) tasks, groups of experts do often significantly disagree with each other^5,7,8^. In 2021, Kahneman et al.^9^ published a major contribution to this topic called *Noise: a flaw in Human Judgment*, which convincingly makes the case that fellow experts in many disciplines do differ. These authors^9^ make distinctions between judgments and opinions where with the former, experts are expected to provide a response from a (fixed) set of alternatives, whereas opinions are much more open-ended. In this paper, we deal with tasks that require the various experts to make *judgments*.

There are four main sources of annotation inconsistencies^2,8,10–17^: (a) Insufficient information to perform reliable labelling (e.g., poor quality data or unclear guidelines); (b) Insufficient domain expertise; (c) Human error (i.e., slips & noise); (d) Subjectivity in the labelling task (i.e., judgment & bias). In this study, where highly experienced clinical annotators were used and the labelling task was well understood with 60 instances to annotate, we believe the main source of inconsistency investigated is the interrater variability resulting from observer bias, judgment, and noise. Throughout this paper, we define ‘noise’ as *system noise*, i.e. unwanted variability in judgments that should ideally be identical^9^.

Kahneman et al.^9^ notes between-person noise (i.e., interrater variability) in the medical profession is most common when clinicians are required to make judgments, as opposed to following a routine or largely mechanical diagnosis (i.e., consisting of set tests or quantitative rules); Kahneman et al. outline a series of examples. Jain et al.^18^. found that in diagnosing breast proliferative lesions, agreement amongst pathologists only had a ‘fair’ agreement (Fleiss’ *κ* = 0.34). Regier et al.^19^ showed highly trained specialist psychiatrists only agreed on a diagnosis of ‘major depressive disorder’ 4–15% of the time (Fleiss’ *κ* = 0.28)^20^. Halford et al.^21^ showed minimal agreement among EEG experts for the identification of periodic discharges in continuous ICU EEG recordings (average pairwise Cohen’s *κ* = 0.38). Moor et al.^22^ describe the significant issues of disagreements on the definition of sepsis - a leading causes of death in ICUs worldwide. Zhang et al.^23^ investigate Emergency Department (ED) clinicians’ referrals to inpatient teams and found for 39.4% of the admissions, patients were admitted to a different inpatient team than that initially referred to by the ED. Xia and Yetisgen-Yildiz^24^ showed almost no agreement between clinical annotators identifying pneumonia from chest x-ray reports (Cohen’s *κ* = 0.085), and that “medical training alone is not sufficient for achieving high inter-annotator agreement”. The presence of noise is clearly pervasive across a variety of medical domains, including ICU settings.

Using such clinicians to establish the Knowledge Base results in a ‘shifting’ ground truth, depending on which expert(s) are used. Label noise in training data has been shown empirically to result in^4,11,25–28^: decreased classification accuracy, increased complexity of inferred models (e.g., increasing size of decision trees), increased number of training samples needed, and a difficulty in feature selection. To the best of our knowledge, this paper is one of the first studies that investigates biases/inconsistencies among a sizeable number (11) of clinicians in acute clinical decision-making scenarios (ICU settings), using an external validation dataset.

Frequently, two approaches are used to address class label noise in ML development. The first involves utilising data cleansing methods, where noisy labels are identified and relabelled/removed before training. The second involves using label noise-tolerant algorithms, where label noise is accounted for during learning^10,12,29^. Moreover, applying these methods may result in the loss of subtle and potentially important differences between annotators’ class labels. (This latter issue is addressed in the Further work section). There is some informative literature discussing methods to improve the quality of clinical labels, including establishing clear annotation guidelines^24^ and modelling annotation errors of the human experts^30^. However, most of this literature considers image classification tasks – there is a lack of empirical studies around improving the quality of symbolic labels within medical annotation tasks.

The aim of this study is to assess the (in)consistency of human annotations for AI model development and the impact on real-world clinical decision-making in ICU settings. The overall class label quality is strongly impacted by disagreements between annotators. The focus of this study is on investigating the impact and effective utilisation of experts’ disagreements (via their annotations) in developing ML models rather than resolving the deviation of their judgments for forming a “ground-truth”. We conduct extensive experiments demonstrating how differences in judgments between clinical expert annotators may lead to classification models with varying performance (therefore varying clinical utility), and how to obtain an optimal consensus from such differences, to facilitate AI driven clinical decision-making. Specifically, Sleeman et al.^5,7^ reported clinical experts sometimes disagree when labelling the severity of an Intensive Care Unit (ICU) patient on a five-point scale (A-E), based on the values of six clinical variables. The current study addresses the question: ‘What are the implications of these differences in judgment on the resulting classifier model performance and real-world ICU clinical decision-making?’ We therefore proposed the hypothesis that the *M* classifiers, derived from datasets individually labelled by *M* clinical experts, produce consistent classifications when applied to a relevant external dataset. The objectives of this study are to: 1) Build classifiers from the 11 individually annotated Queen Elizabeth University Hospital (QEUH) ICU datasets. 2) Evaluate the classifiers’ performances on real-world discharge outcomes (discharged alive from ICU and died in ICU) in an external ICU dataset: HiRID. 3) Assess various approaches for dealing with annotation inconsistencies, as these frequently create sub-optimal AI models.

## Results

This study focuses on a scenario of using AI technologies for facilitating a clinical decision-making problem that ICU consultants encounter on a day-to-day basis, as described below.

### Clinical question

**Fig. 1:**
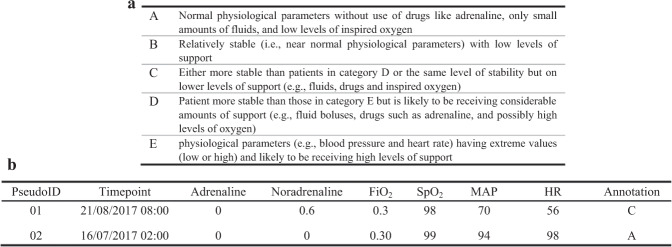
Description of the QEUH annotated training data. **a** ICU-PSS annotation categories. **b** Example instances of a QEUH ICU annotated dataset.

Can we use a five-point ICU Patient Scoring System (ICU-PSS) scale (A-E) to address the question “How ill is the patient?”, where E represents severe cardiovascular instability, and A represents a relatively stable patient. Figure 1a provides a description of the ICU-PSS scale and Supplementary Table 1 contains further details.

The training dataset was obtained from the Glasgow Queen Elizabeth University Hospital (QEUH) ICU patient management system. It contains 60 data instances described by six clinical features: two drug variables (Adrenaline and Noradrenaline) and four physiological parameters (FiO_2_, SpO_2_, mean arterial pressure (MAP) and heart rate (HR)). Note, the six variables are those which clinicians regularly use in the ICU to assess how ill a particular patient is. Example annotations are shown in Fig. 1b. The QUEH dataset may contain trauma and non-trauma ICU patient data.

Our main aim is to assess the (in)consistency of human annotations for AI model development and the impact on real-world clinical decision-making in ICU settings. This is broken down into the following aspects.

i. Evaluation setup: (a) ML models are developed using the QEUH annotated datasets; (b) external validation datasets are prepared, and all model performance assessments are to be conducted on these datasets.
ii. Consistency quantification: We choose Cohen’s *κ* scale^31,32^ and Fleiss’ *κ*^33,34^ to measure the extent to which annotators’ AI models assign the same category to the same instance. Higher values on these scales suggest stronger levels of agreement. Cohen’s scale can be summarized as: 0.0–0.20 (None); 0.21–0.39 (Minimal); 0.40–0.59 (Weak); 0.60–0.79 (Moderate); 0.80–0.90 (Strong); > 0.90 (Almost Perfect).
iii. Impact on real-world decision-making: we chose two real ICU decision-making scenarios, both of which are binary classification tasks. First, whether a patient should be discharged from ICU in the next hour; second, whether a patient is going to die in ICU within the next hour. We investigate two methods of external validation – one using hourly snapshots of patient data (i.e., static data) and another using time series data (i.e., temporal data).
iv. Evaluate current “best practices” of obtaining the gold-standard: we evaluate (a) whether there is a “super expert” whose judgment should be used as the gold-standard when disagreements occur; (b) whether a consensus can be obtained from all expert judgments to achieve the gold-standard?

**Fig. 2:**
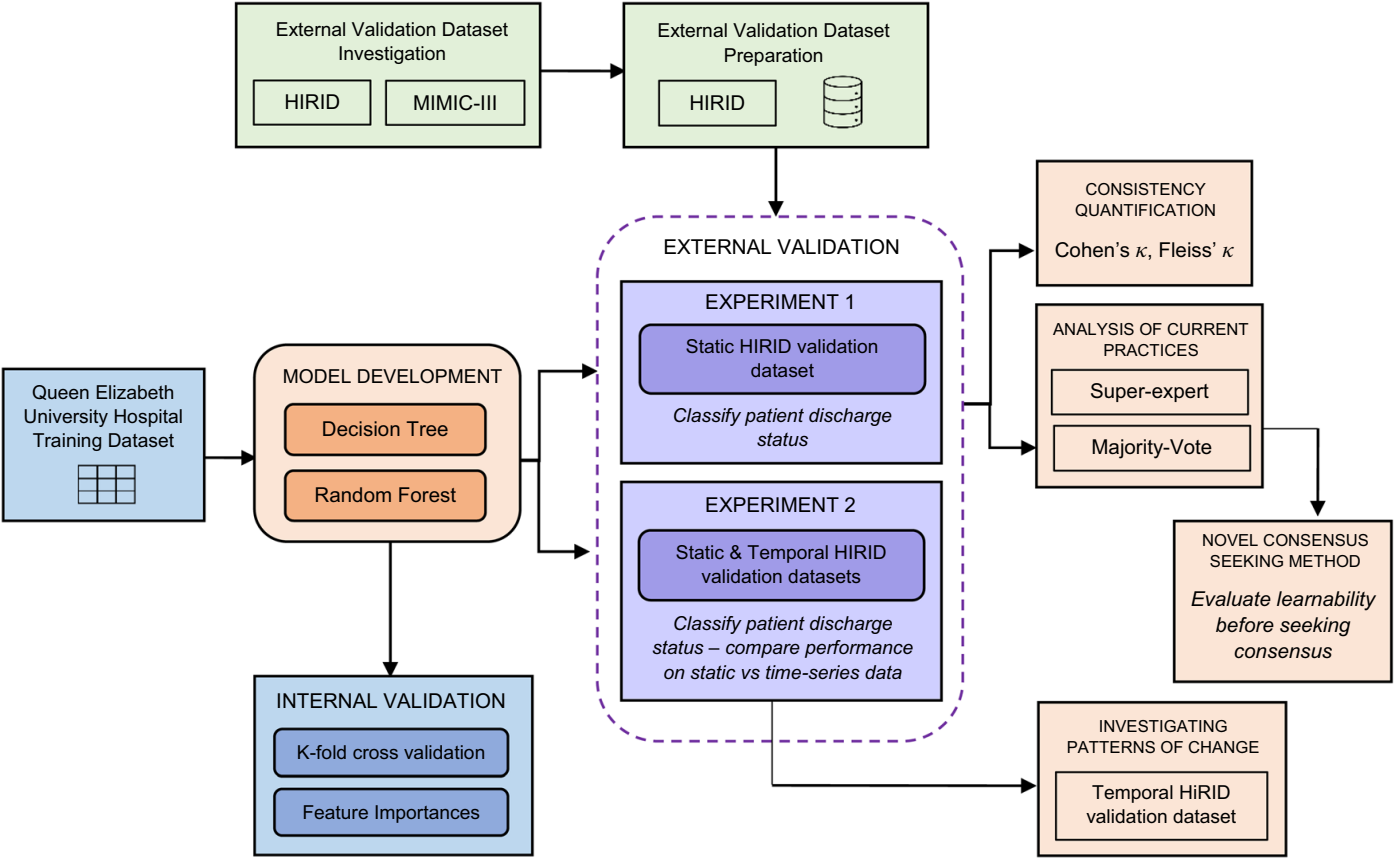
Overview of the experimental approach, outlining the dataflow and key analytical steps. The left component (with three boxes) illustrates the model derivation including dataset, models and internal validation methods. The top component with two green boxes denotes the external validation dataset selection and preparation. The middle component (circled by a dashed line) shows the external validation experiments. The right component (with four pink boxes) describes the external validation experiment details including inconsistent measurements, consensus seeking methods and decision making considering changing patterns.

An overview of the experimental approach described above is found in Fig. 2.

### Quantifying the consistency of expert judgments

Recall that the central hypothesis for this study is: the *M* classifiers, derived from the datasets individually labelled by *M* clinical experts, produce identical classifications when applied to a relevant external dataset.

Decision tree (DT) and random forest (RF) classifiers were built from the QEUH annotated datasets, in part as both are popular choices in clinical machine learning literature. DT was selected as the resulting tree plots can be used to infer the decision-making process of the learnt models, as well as compare the different complexities between annotator models. RF was used to compare whether more powerful models (compared to DT) would make the inconsistency less significant – which we show in later subsections is not the case.

**Fig. 3:**
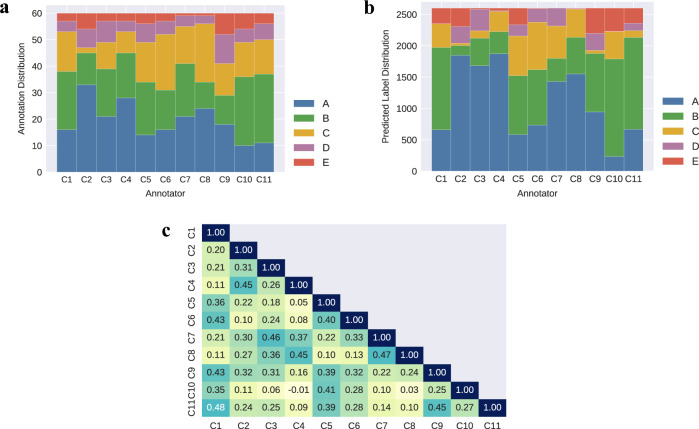
Distributions of the 11 consultants’ annotations on the training dataset and predicted labels on the external validation dataset. **a** Annotation distributions across all consultants’ (C1-C11) labelled QEUH training datasets. **b** Predicted label distributions across the consultants’ RF multiclass models, run on the HiRID validation dataset. **c** Pairwise Cohen’s κ values across all consultant pairs for the predicted labels made by the multiclass RF models on the external HiRID validation dataset.

11 classifiers were derived from each of the 11 consultants’ annotated datasets, which contained data for 6 clinical variables (Adrenaline, Noradrenaline, FiO_2_, SpO_2_, MAP, HR) and the severity class labels (A-E). The annotation labelling (A-E) across the 60 training instances differs across the 11 annotators, as shown in Fig. 3a. Note, we tried class-balancing techniques to balance the class labels within the annotated datasets prior to training, however this did not result in a significant performance difference (see Supplementary Table 2). Therefore, we decided to build classifiers using the original annotated datasets. The 11 consultants who annotated the QEUH datasets were randomly assigned anonymous code names (C1-C11) following the annotation exercise in the previous Sleeman et al.^5^ study. These code names are referred to throughout this paper. Each consultant’s corresponding RF classifier is referred to as C*n*-RF, where *n* refers consultants 1–11.

**Fig. 4:**
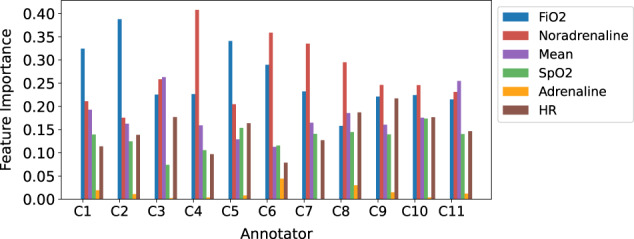
Feature importance distributions across the Random Forest models, trained on the 11 consultants’ (C1–C11) QEUH annotated datasets. The *x*-axis lists the 11 classifiers and the *y*-axis is the importance value with a range from 0 to 1, where 1 denotes the largest importance.

**Fig. 5:**
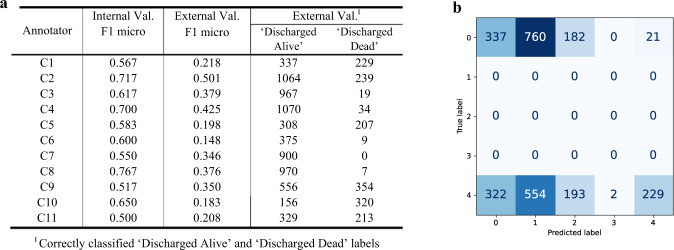
Comparison of internal and external validation performances of the RF models across all 11 consultants (C1-C11). **a** Internal and external validation performances of the consultants’ RF models. For each classifier, the number of correctly classified “Discharged Alive” and “Discharged Dead” labels on the HiRID external dataset are reported. **b** External validation confusion matrix plot for Consultant 1, showing the HiRID dataset true labels and RF model predicted labels across the five classes (A-E): 0 = ICU-PSS label ‘A’, 4 = ICU-PSS label ‘E’.

The trained models predict ICU-PSS labels (A-E) for a patient, indicating their level of severity. A standard internal validation experiment across multiple annotated datasets involves first establishing a ground truth, most likely through taking a majority vote across all annotators for each instance. Then each trained consultant model would be run against this ground truth to establish internal validation performance. We developed and utilised a different method, more relevant to this study, where each trained model was run against the original annotations it learnt from – thus, these internal validation results indicate the ‘learnability’ of the original annotated datasets, i.e., how well the associations between the attribute variables and provided annotations can be learnt, and in turn how easily the annotator’s decision-making can be reproduced. These internal validation F1 (micro) score ranges between 0.50 to 0.77 across the 11 RF classifiers, as seen in Fig. 5a. The feature importance across the six predictive variables differs across the classifiers, as shown in Fig. 4.

With all the external validation experiments, the focus is on predicting the two extreme clinical scenarios (discharged alive from ICU or died in ICU). In this first external validation experiment, the trained models were run on a HiRID test dataset, to predict severity labels (A-E) on 2600 instances containing data for the same 6 clinical variables (1300 of these instances corresponds to patients who are discharged alive from that ICU, and a further 1300 patients who died in that ICU). As our focus is a binary (discharge status) classification task, we mapped the multiclass A-E severity label classifications to binary discharged/died classifications as follows:

- In the last hour before a patient is discharged (alive) from ICU, their classification on the ICU-PSS scale is ‘A’.
- In the last hour before a patient dies in ICU, their classification on the ICU-PSS scale is ‘E’.

Note, in the HiRID dataset, not all patients with an ‘A’ classification were discharged within the next hour. Similarly, not all patients with an ‘E’ classification died within the following hour; many patients upon arrival to ICU are extremely ill and are often rated as an ‘E’.

The predicted labels across the 2600 HiRID test instances differ across the annotators, as shown in Fig. 3b. It is clear from reviewing this diagram that there is a great deal of variation in the classifications of the experts’ models, with only a few models having comparable labels. The corresponding pairwise inter-annotator agreements (IAAs) for these A-E predicted labels, using Cohen’s scale, range between −0.01 (Low/None) to 0.48 (Weak) across the annotator models, and are shown in Fig. 3c. The average pairwise Cohen’s κ score is 0.255 (Minimal agreement). Fleiss’ κ for these predicted labels is 0.236 (Fair agreement). Note, IAA is used as an abbreviation for “Inter-Annotator Agreement” throughout this paper.

These results were obtained using the Random Forest classifiers^35^, trained on the 11 consultants’ annotated datasets. The corresponding classifiers obtained using the Decision Tree algorithm^25^ gave comparable results, see ref. ^36^. Classifiers trained using XGBoost and SVM also gave comparable results to the RF models, as shown in Supplementary Fig. 3.

### Investigating inter-annotator agreement across the ICU discharge status classifications

Further, we consider the actual decisions which the classifiers from the 11 QEUH consultants made concerning the HiRID validation dataset which you will recall, contained 1300 instances which correspond to the patient being discharged alive in the next hour (i.e., ICU-PSS label ‘A’, as outlined in the mapping above) and 1300 instances where the patient died in the ICU within the following hour (i.e., ICU-PSS label ‘E’). These results are summarised in Fig. 5a. Recall, the trained classifiers predict ICU-PSS classification labels (A-E) for a patient, indicating their level of severity. In this first external validation experiment, we treat the trained models as predicting three classes: CL1 = A, CL2 = B/C/D, & CL3 = E. The external validation F1 scores reported in Fig. 5a are calculated using the F1 micro average – computing a global average F1 score by counting the sums of the True Positives, False Negatives, and False Positives. F1 score^37^ is the harmonic mean of the precision and sensitivity of the classifier, where a higher score indicates a higher performing model.

Figure 5a reports the number of correctly classified “Discharged Alive” and “Discharged Dead” labels across all 11 classifiers. These results suggest that C10 is the ‘most reluctant’ to discharge patients, with the lowest number of correct “Discharged Alive” classifications, referring to the number of correctly predicted admissions discharged alive within 1 h. In contrast, C2 and C4 are the ‘most likely’ to discharge patients, with the highest number of correct “Discharged Alive” cases.

#### Scenario 1: Patients discharged alive from ICU

Focusing only on the instances where the patient was discharged alive, we observe the average pairwise inter-annotator agreement (Cohen’s *κ*) is 0.21 (Minimal agreement). Fleiss’ *κ* for these predicted labels is 0.174 (Slight agreement).

#### Scenario 2: Patients died in ICU

Focusing now on the instances where the patient died in ICU, we observe the average pairwise inter-annotator agreement (Cohen’s *κ*) is 0.28 (Minimal agreement). Fleiss’ *κ* for these predicted labels is 0.267 (Fair agreement).

This suggests clinical domain experts agree more when predicting mortality, compared to making discharge decisions. Note, due to the low number of ‘E’ labels across the annotated datasets, limited insights and comparisons can be inferred for these predicted “died” labels. In future related studies we will acquire more class-balanced datasets to address this issue.

Figure 5b shows an example one consultant’s (C1) confusion matrix plot, outlining the distribution of the RF predicted labels when run on the HiRID validation dataset. Predicted labels 0–4 correspond to ICU-PSS labels A-E, respectively. True label = 0 corresponds to the patient being discharged alive from ICU within the next hour (i.e., ICU-PSS label ‘A’); and true label = 4 corresponds to the patient having died in ICU within the following next hour (i.e., ICU-PSS label ‘E’). This confusion matrix shows C1-RF correctly classified the patient as ‘Discharged Alive’ for 337 cases, and correctly classified the patient as ‘Discharged Dead’ for 229 cases. The trained models were treated as predicting three classes: CL1 = A, CL2 = B/C/D, & CL3 = E.

As the QEUH training data consists of hourly snapshots of patient physiological/pharmacological readings, we ran this external validation experiment with a HiRID validation dataset containing similarly static data. However, Fig. 5a shows the external validation performance is significantly lower than the internal validation performance. This could indicate that extreme decision-making at ICUs (predicting discharge/death) may require continuous monitoring (i.e., using time series data) – this is explored further in the later subsection ‘Assessing Time Series External Validation Methods’. Additionally, the annotation distributions shown in Fig. 3a suggest that human annotators may be less likely to choose extreme label categories (i.e., A or E) when presented with a multiclass labelling task, which in turn results in poor performance when predicting these scenarios.

**Fig. 6:**
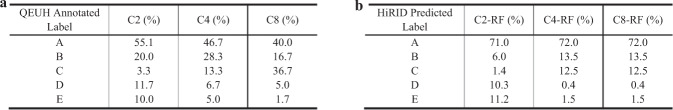
QUEH annotations across the highly learnable expert-labelled datasets and resulting RF predicted label distributions. **a** Annotation distributions across the QEUH labelled datasets for C2, C4 & C8. **b** Predicted label distributions generated by classifiers C2-RF, C4-RF & C8-RF when run on the HiRID validation dataset.

For the classifiers that had high internal validation performance (C2-RF, C4-RF, C8-RF), we can infer that these consultants’ annotated datasets were highly learnable (recall, ‘learnability’ indicates how well the associations between the input variables and provided annotations can be learnt, and in turn how easily the annotator’s clinical rationale can be reproduced). Despite having similarly high internal validation performance, consultants C2 and C8 differ in their initial QEUH annotation distributions and subsequent feature importance distributions, as outlined in Fig. 3a and Fig. 4, resulting in differing distributions in their predicted labels on the HiRID validation dataset. As shown in Figs. 6a and 6b, the C2 QEUH annotated dataset consists of 3.3% ‘C’ labels and 10.0% of ‘E’ labels, whereas the C8 annotated dataset consists of 36.7% ‘C’ labels and 1.7% ‘E’ labels. The inferred C2-RF classifier predicted labels consists of 1.4% ‘C’ labels and 11.2% ‘E’ labels, whereas the inferred C8-RF classifier predicted labels consists of 12.5% ‘C’ labels and 1.5% ‘E’ labels. Overall, the C2-RF and C8-RF classifiers have minimal agreement across their classifications when run on the HiRID dataset (pairwise Cohen’s *κ* = 0.27).

### Analysis of current practices on obtaining Gold-standard

In this subsection, we evaluate two types of best practices in obtaining gold-standard from multiple domain experts:

(a) Super expert: use a more senior annotator’s labels or use decisions from an adjudicator when disagreements happen; (b) Majority-Vote: Seek consensus from **all** different judgments as the ground-truth^38–40^.

Regarding the “super expert” assumption, we could not make this assessment directly, as we do not know which annotators are more senior, due to the anonymization of the dataset. To work around this, we use the correlation between internal and external model performances as a proxy indicator. This is because, if the super-expert assumption holds, one could assume that models with higher (or lower) performance internally are likely to have higher (or lower) performances in external validations. Figure 5a lists the internal and external validation results. The Pearson correlation between the two results is 0.51, meaning they are not strongly associated. The results of this analysis suggests that the super-expert assumption, i.e., that the gold-standard can always be provided by the most senior colleague, is not always true. We observe that even the well performing models in internal validation do not perform as well in external datasets (e.g., C4-RF and C8-RF). In fact, the initial annotations of the QEUH dataset shows similar levels of disagreement amongst the consultants as shown on the HiRID validation dataset. As we show later, a superior model can often be achieved by considering diverse judgments in a selective majority-vote approach.

Additionally, we investigated taking a consensus of all experts’ annotations (a common practice). Figure 5a shows the varied internal validation performance across the QEUH datasets, indicating a difference in learnability across the 11 annotated datasets. The models with higher internal validation performance indicate easier learnability (e.g., C8), which potentially reflects more consistent annotation rules and a simpler decision-making process. Models with lower internal performance indicate a poorer learnability, with potentially less consistent / more complex classification rules (e.g., C7).

**Fig. 7:**
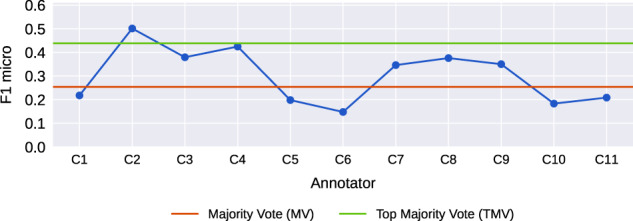
Multiclass random forest QEUH models, run on the HiRID external validation dataset. Majority Vote (MV) refers to a total majority-vote consensus model. Top Majority Vote (TMV) was built from the majority-vote labels across the top-performing consultant models.

To assess the reliability of taking a consensus, we compared the external validation performance of a consensus Majority Vote (MV) model, built from the majority-vote labels across all 11 annotated datasets, to a Top Majority Vote (TMV) model, built from the majority-vote labels across the top-performing consultant models (where internal validation F1 micro > 0.7). Figure 7 shows TMV (F1 micro = 0.438) performs significantly better than MV (F1 micro = 0.254). In fact, TMV outperforms almost all the consultant models. This indicates it is important to assess learnability of each domain expert’s judgments before creating a consensus, because poorly learnable (expert) judgments often lead to poor performances.

### Assessing Time-series external validation methods

After further discussion with ICU professionals, we established ICU consultants’ clinical decision-making commonly considers the trend in patient physiological and pharmacological parameters across the period of time prior to assessment (e.g., across the previous 5–10 h). We, therefore, incorporated a time-series component into this second external validation experiment and investigated how this impacts the performance of the QEUH classifiers. We believe this experiment is a more clinically relevant assessment of the expert models, as it provides the more realistic task of classifying discharge status given patient parameter readings over a period of time (rather than a single snapshot).

Within this second external validation experiment, we compared the performance of DT classifiers, trained on the QEUH annotated datasets, on both static and temporal HiRID datasets. The static HiRID validation dataset contains 1064 records (of 1064 unique patients), where all data instances are readings within 1 h before the patient is discharged alive (i.e., ICU-PSS label ‘A’) or within 1 h before the patient died (i.e., ICU-PSS label ‘E’). The temporal HiRID validation datasets contain 5320 records (across the same 1064 unique patients), made up of five records per patient – one reading for each of the 5 h before discharge/death.

To assess the performance of the trained DT classifiers on the temporal validation datasets, for each patient timepoint the weighted sum of the five (hourly) ICU-PSS predictions was calculated, and a mean value was obtained (resulting in 1,064 severity classifications within the temporal datasets). These A-E predicted labels were treated as a 1–5 ordinal scale, therefore the weighted sum values were all in the range 1–5. Again, the trained models were treated as predicting three classes: CL1 = A, CL2 = B/C/D, & CL3 = E. We explored two methods of mapping the weighted sum values (1–5) to these three classes, with differing cut-offs, as shown below. Further details are outlined in the Methods section.

i. ‘Extreme’: CL1 = 1, CL2 = > 1–4, CL3 = > 4.
ii. ‘Neutral’: CL1 = ≤ 3, CL2 = > 3-<4, CL3 = ≥ 4.

**Fig. 8:**
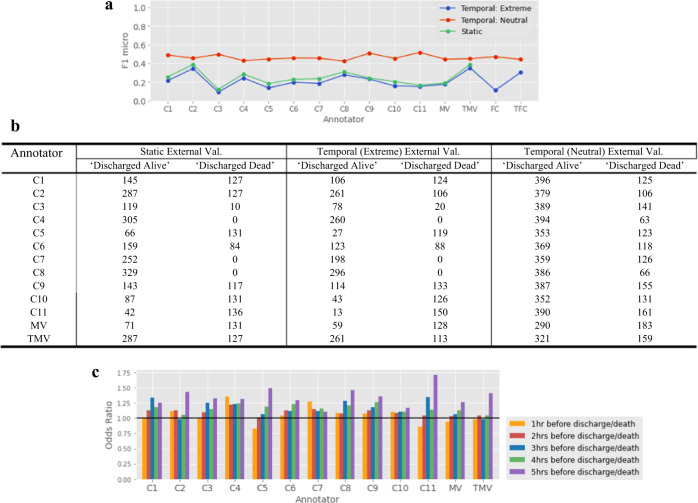
Additional analyses on a HiRID time-series validation dataset. **a** Comparison of external validation performance on static vs temporal HiRID validation datasets. **b** Correctly classified ‘discharged alive’ and ‘discharged dead’ labels made on static and temporal HiRID external validation datasets. **c** Odds ratio distributions of the Logistic Regression model trained on HiRID predicted labels 1–5 h before discharge/death.

Within this experiment, in addition to the MV and TMV consensus models, an additional ‘Fuzzy Consensus’ (FC) model was built. This FC model was built by combining the individual models’ outputs by considering their outputs as confidence values for the binary classification task on the temporal external validation datasets (discharged alive vs died). We treated the A-E predicted labels as predictions on a 1–5 ordinal scale (i.e., A = 1, B = 2, C = 3, D = 4, E = 5). In this scale, A represents discharged alive within the next hour, and E represents died within the following hour. Within this consensus method, all predictions are captured and interpreted as ‘fuzzy’ labels^41^ in calculating the overall discharge status prediction for each patient. For each hourly prediction, per patient, the model outputs (1–5) were averaged, but excluding any ‘3’ (i.e., ‘C’) predicted labels in this calculation. ‘3’ is excluded as this confidence value sits directly in the centre of the 1–5 scale and is therefore interpreted as “uncertain”. Following this averaging calculation, for each patient timepoint the weighted sum of the five (hourly) ICU-PSS predictions was calculated, using both the ‘Extreme’ and ‘Neutral’ cut-offs outlined above. The results are shown in Fig. 8a. Further details around the FC model calculation are found in the Methods section. A ‘Top Fuzzy Consensus’ (TFC) model was also built from the majority-vote labels across the top-performing consultant models (where internal validation F1 micro > 0.7).

Figure 8a shows all annotator models perform better on the temporal (neutral) validation dataset, compared to the temporal (extreme) and static datasets. The models have higher performance on the dataset generated with more neutral classification mapping, compared to the more extreme mapping, as the extreme mapping method excludes a much greater number of patient datapoints from the CL1 and CL3 classes (recall, only CL1 and CL3 classes are present in the HiRID validation datasets). An additional mapping was investigated using the following cut-offs: CL1 = ≤ 2, CL2 = > 2-< 4, CL3 = ≥ 4, see Supplementary Fig. 1 for these results.

As shown in Fig. 8a, we observe that the well performing models in internal validation (C4-RF and C8-RF) do not perform as well when run on the external temporal external datasets. The Pearson correlation between the internal validation results and temporal (extreme) external validation results is 0.64, meaning they are not strongly associated. Similarly, the Pearson correlation between the internal validation result and temporal (neutral) external validation results is −0.51. This provides some further evidence that the super-expert assumption may not always hold in acute clinical settings.

Figure 8a shows the Top Majority-Vote model (TMV) performs significantly better than the consensus Majority-Vote model (MV) on the static validation dataset, as observed in the previous experiment. TMV also performs significantly better than MV on the temporal (extreme) dataset and slightly higher than MV on the temporal (neutral) dataset. This further suggests the importance of assessing learnability of domain experts’ judgments and excluding the poorly learnable expert annotations before obtaining a consensus as ground-truth. The Top Fuzzy Consensus (TFC) model also performs well – indicating consensus is consistently improved after selecting models based on the individual models’ learnability.

Figure 8b reports the number of correctly classified “Discharged Alive” and “Discharged Dead” labels across all 11 classifiers, run on the static and temporal HiRID validation datasets.

#### Scenario 1: Patients discharged alive from ICU

Focusing only on the instances where the patient was discharged alive, we observe the average pairwise IAA, i.e., Cohen’s *κ*, is 0.239 (Minimal agreement) on the temporal (extreme) dataset, where Fleiss’ *κ* for these predicted labels is 0.211 (Fair agreement). When run on the temporal (neutral) dataset, average pairwise IAA is 0.284 (Minimal agreement) and Fleiss’ *κ* is 0.294 (Fair agreement).

#### Scenario 2: Patients died in ICU

Focusing now on the instances where the patient died in ICU, we observe the average pairwise IAA is 0.327 (Minimal agreement) on the temporal (extreme) dataset, where Fleiss’ *κ* for these predicted labels is 0.326 (Fair agreement). When run on the temporal (neutral) dataset, average pairwise IAA is 0.587 (Weak agreement) and Fleiss’ *κ* is 0.579 (Moderate agreement). This further indicates that clinical domain experts may agree more when predicting mortality, compared to making discharge decisions.

We conducted additional analysis to investigate how supervised-learning models perform on classifying patient discharge status, after training on the predicted labels (A-E) generated (by the DT classifiers) on the temporal HiRID dataset. This involved training decision tree and logistic regression (LR) models on each consultant’s DT classifier predicted labels (A-E) across the five hours before discharge/death for each patient (i.e., 5 predictor features), see Supplementary Fig. 2.

The odds ratio distributions indicate the difference in weightings (i.e., importance) across the five hourly variables, in making the patient discharge status classification (discharged alive or died). The predictions at 5 h before discharge/death were most important in the LR model discharge status classification across most consultant models, as well as for MV and TMV. For a majority of models, the predictions at 1 h before discharge/death were the least important in making the final discharge status classification, which is notable as this contradicts an intuitive hypothesis that discharge predictions closer to time of discharge/death are indicative of final discharge status.

## Discussion

This study focussed on assessing the disagreements between clinical annotators and evaluating the impact of these disagreements on the performance of resulting ML models, within ICU settings. In particular, we evaluated current ‘best practices’ of seeking consensus, and our results suggest these may not work well in acute clinical settings. Our analysis points out a novel, more reliable approach - evaluating learnability before seeking consensus.

The varied label classifications shown in Fig. 3b and the low pairwise agreement in Fig. 3c (average Cohen’s *κ* = 0.255 i.e., Minimal agreement) are sufficient to reject the central hypothesis – concluding that the classifiers, derived from datasets individually labelled by the 11 clinical experts, do **not** produce consistent classifications when applied to a relevant external dataset. Further analysis on two ICU decision-making scenarios showed inconsistency varies in different situations: these clinical domain experts seem to have higher agreement on more critical situations like predicting mortality.

A deep dive on assessing the current practices in obtaining ground-truth makes two actionable suggestions: (a) super experts (who are more reliable than everyone else) may not exist in acute clinical settings, and diverse responses might be more reliable for obtaining optimal models; (b) learnability (quantified as internal validation performances) is a key metric to be assessed on domain experts’ judgments, and avoiding poorly learnable judgments might lead to better ground-truths, therefore better external validation performances.

Further to point b), an approach to detect and exclude experts who inconsistently apply their annotation rules is summarised as follows: All experts are to annotate the same set of (training) instances; from these annotated datasets a classifier would be inferred for each expert. The ‘learnability’ of each classifier is obtained through an appropriate method e.g., k-fold cross validation, where the trained models are run on their original annotations – this is a check for consistency of each expert’s annotation rules. Then exclude all models that do not perform above a predefined threshold (i.e., the models built from annotated datasets with low learnability). The results shown in Figs. 7, and 8a indicate that this method can be applied to utilise disagreements between clinical annotators in generating higher performing consensus models (i.e., TMV and TFC).

After excluding poorly learnable models, we observe there can be significant differences in the classifications made by the distinct expert models (e.g., C2-RF and C8-RF), as outlined in the Results section. This agrees with the observation made by Welinder et al.^42^ that some annotators tend to be more extreme in their labelling, whereas others are more moderate. As classifiers C2-RF and C8-RF were inferred from learnable annotated datasets (indicated by good internal validation performance), this suggests the differences in resulting predicted label distributions may stem from differences in consultant judgments. Therefore, these may be valid and clinically useful differences we may not want to ignore. Current ML approaches to addressing label noise in training datasets include data cleansing (i.e., removing the noisy labels) or utilising noise-robust/noise-tolerant algorithms. Applying these methods may result in losing the useful granular differences between annotator judgments. Additionally, applying the majority-vote or the top-majority-vote approach (described in Results section) can result in a loss of subtle differences between annotator judgments. This issue is to be addressed in the Further work section.

### Further work

Most of the classifiers built in this study have poor internal and external validation performance, reflecting poor real-world decision-making. However, this poor performance could be due a variety of reasons: small/unbalanced training dataset, selected features are not the most predictive, the need to assess patients on multiple timepoints, differences between ICU settings etc. A similar study using a larger set of annotations, with more balanced classes (and possibly more/different features), is needed to further investigate the characteristics of this real-world non-random noise and obtain more reliable results for the implications on model performance, as well as the effectiveness of our proposed consensus seeking method (i.e., evaluating learnability before seeking consensus). This should include a very large cohort of clinical annotators from a sizable number of UK ICUs, to provide a diverse set of judgments, as well as multiple external validation datasets from different countries, to assess how models perform across different settings.

Using these larger annotated datasets, further analysis should be conducted around the reasonings behind the inconsistencies across annotators, e.g., bias, judgments, noise, limited feature selection - as well as ways to resolve these. This should involve analysing the way in which consultants disagree, including the characteristics of easy cases (high agreement amongst annotators) and hard cases (high disagreement amongst annotators). Additionally, studies aimed at reducing the levels of disagreements between (clinical) experts by enhancing the description / presentation of the labelling task(s) should be considered^5^.

Following the findings discussed, further research to detect and investigate expert intra-annotator consistency is planned. Intra-annotator consistency can be detected easily by including repeated items in datasets to be annotated – following this, inconsistent experts can be removed from further analyses. Additionally, we will explore further whether removing the ‘poorly learnable’ annotated datasets prior to training increases inter-annotator agreement and produces better external validation results, as well as more consistent decision-making. If found to be true, this would verify that assessing learnability of individual expert judgments is an important step in training ML models, meaning current practices of seeking consensus directly from all available expert judgments to obtain ‘gold-standard’ need to be revised - as poorly learnable judgments may cause issues in reaching a true gold-standard.

Additionally, in practice, people tend to trust highly experienced (“super”) experts more, hence, their judgments play more important roles in obtaining a ‘gold-standard’. Further investigation is needed to confirm whether ensemble models perform better than individual experienced domain experts.

Moreover, in a further study, the annotation task could be modified by requesting each consultant to assign a confidence factor, between 0–1, to each of their annotations. Additionally, skill level (based on years of experience or specialty) of each annotator can be captured. These could then be used as weighting factors during model training, reducing the effect of low confidence labels and increasing the contribution of higher skilled experts in calculating the consensus. Further, these confidence values will facilitate analysis around easy/hard cases. Nettleton et al.^41,43^ have conducted extensive experiments utilising weighting and confidence factors in capturing responses.

If the ML decision-support system is thought to be a safety critical one, then it is vital to include some further analyses to establish which distinct expert classifier(s) to use. For example, run each of the classifiers against a set of task-solution pairs pre-specified by an expert panel and eliminate those classifiers / experts who correctly solve less than a pre-defined percentage. The effectiveness of such filters depends critically on the instances chosen by the panel. This, however, is an appropriate approach to use when working in (safety-critical) areas where the differences between two (or more) classes are slight, but where the consequences of a misclassification are high. This approach has been used extensively in IBM’s Jeopardy System^44^ and earlier in the KRUST system^45^. (This step should be run as well as the statistical / numerical ones discussed earlier.)

Figure 8c provides a very interesting insight, namely that the predicted severity labels (A-E) at 5 h before discharge/death were most important in the LR model discharge status classification across most expert models, whereas the predictions 1 h before discharge/death were least important – a somewhat counterintuitive finding. Further research is needed here, in collaboration with ICU professionals, to investigate how the trends in physiological readings across a period before discharge/death can be used to inform discharge status predictions.

## Methods

### Experiment design

This study is focussed on simulating a real-world ICU decision-making scenario, where disagreements are fairly common and unavoidable, and investigating the impact of these clinician disagreements on resulting machine-learning models. To achieve this aim, all aspects of the experimental approach (outlined in Fig. 2) were carefully considered. The main factors are discussed below.

The Queen Elizabeth University Hospital training dataset consists of 60 instances of ICU patient data, across 6 descriptive variables. As disagreements are common across clinicians (reasons are multifactorial and summarised in the Introduction section), in order to minimise the intra- and inter-inconsistency across annotators, we selected a simple classification task consisting of a limited set of features and data instances. The annotation task selected for the basis of this research was therefore clinically relevant, but more research focussed – allowing the clinicians’ decision-making process to be correctly captured.

The ICU PSS scale (developed in the period 2000–2005)^46^ allows clinicians to make judgments of a patient’s status, at particular points in time, on the basis of a limited number of six descriptors. There are many situations in Medicine where decision / judgments must be made based on partial information – it is this scenario which this paper addresses. The ICU-PSS scale has five annotation categories which, although categoric, can be viewed as confidence scores of each annotator about the patient’s severity status (where A = more stable likely to be discharged soon and E = very unstable patient requiring significant pharmacological support). This can A-E confidence scale can therefore be applied to a binary external validation task, as discussed in the ‘Assessing Time-series External Validation Methods’ subsection. Further, this ICU-PSS scale is simpler and easier to understand compared to alternative clinical scoring tools (e.g., SOFA^47^), resulting in a simpler classification task which allows each clinician’s decision-making/annotation rules to be better captured and compared.

The six clinical variables were selected, and the five-point qualitative description of ICU patients (A-E) was developed, in conjunction with several ICU specialists in a previous study. The four basic physiological parameters (FiO_2_, SpO_2_, Mean arterial pressure, Heart Rate) are used by clinicians as indicators of any appreciable improvement or deterioration in patient condition. The drug fields (Adrenaline and Noradrenaline) indicate the amount of pharmacological support required by the patient. A detailed description of these ICU-PSS categories is found in Supplementary Table 1.

There are multiple noise-tolerant ML classification algorithms^10,12^, that can address the issues of label noise during learning. In this study, decision tree (DT) and random forest (RF) classifiers were more appropriate selections, in part because both widely used in clinical settings. More importantly, DT was selected as the resulting tree plots can be used to infer the decision-making process of the learnt models, as well as compare the different annotation rules and complexities between annotator models. RF was used to compare whether more powerful models would make these inconsistencies less significant (which we have shown is not the case).

To compare the consultants’ model performances external model validation was carried using HiRID validation datasets. The QEUH classifiers were built to predict judgments on a 5-point A-E ICU-PSS scale. However, the HiRID validation datasets focused on a binary classification task of predicting discharge/death in the next hour (i.e., A or E values on the ICU-PSS scale). The HiRID database does not contain ICU-PSS ground truth values, nor similar multi-class severity ratings. Therefore, the ground truth discharge status was selected as the validation classification task since the ICU-PSS A-E is comparable to a confidence score for the patient discharge status (where A = discharged alive within 1 h and E = died within 1 h). As the focus of this study is investigating the impact of clinical annotator disagreements on model performance, rather than on improving label quality/model performance, the difference between initial annotation task and model validation task has minimal impact on experiment findings.

### QEUH training dataset

The Glasgow Queen Elizabeth University Hospital training data is de-identified. The 60 instances were randomly selected from a pool of 80,291 hourly patient records obtained from the QEUH patient management system (containing data from trauma and non-trauma patients).

Note, no ground-truth severity or discharge status data for the patients in this QEUH dataset was captured in the previous Sleeman et al.^5^ study. This data could not be later retrieved due to the anonymisation of the patients.

### Class-balanced training datasets

We investigated class-balancing methods to balance the class labels within the annotated datasets during training, through adding the RandomForestClassifier parameter class_weight = balanced. This did not result in a significant performance difference, compared to using the original annotated datasets. The internal and external validation results with this balanced class weight condition are outlined in Supplementary Table 2.

### Internal validation

Internal validation metrics were obtained through 5-fold cross validation, utilising the full training dataset. Each trained model was run against the original annotations it learnt from – thus, these internal validation results indicate the ‘learnability’ of the original annotated datasets, i.e., how well the associations between the attribute variables and provided annotations can be learnt, and in turn how easily the annotator’s decision-making can be reproduced. Figure 5a shows the performance of the optimal RF model for each of the 11 consultant annotators. These models were optimised on F1 micro.

Feature importance distributions, shown in Fig. 4, were obtained using the scikit learn feature_importances_property. This is calculated as the normalised total reduction in node impurity (gini or entropy) brought by the feature. For the models with good internal validation performance (F1 micro > 0.7), the differing feature importance distributions reflect the different rationales and decision-making processes between annotators. For certain annotators (C4), we can infer Noradrenaline is the most important feature when deciding to annotate a label ‘A’ classification. For some (C2), FiO_2_ is most important when making this classification. For others (C10), the rationale is more balanced on Noradrenaline and FiO_2_.

### Investigations & Pre-processing of possible validation datasets: MIMIC-III & HiRID

Broad external validation, using data from similar participants but from a different hospital or country, is considered the gold-standard for reliable estimates of model performance and generalisability/transportability^48–56^. Two external ICU datasets were investigated, namely:

1. **HiRID** (v1.1.1): a freely accessible critical care dataset containing de-identified data for 33,000 ICU admissions to the Bern University Hospital, Switzerland, between 2008 and 2016^57,58^.
2. **MIMIC-III** (v1.4): a freely available database containing de-identified data for 40,000 ICU patients of the Beth Israel Deaconess Medical Centre, Boston, United States, between 2001 and 2012^58,59^.

Both databases contain ICU patient data from a different hospital and country, compared to the Glasgow QEUH training data, thus satisfy the criteria for broad external validation. As the classifiers extracted from the annotated datasets, produced by the QEUH clinicians, contain certain descriptors it was vital to ensure that these are present in the external datasets. Specifically, the following checks were made on the HiRID and MIMIC-III datasets:

i. The datasets contained the same 6 descriptors, and the units associated with each of these variables were either identical or, at least known, so numerical scaling could be applied, if necessary.
ii. Considerable amounts of effort was required to find all the synonyms used in these two datasets for the 6 descriptors used in the QEUH (annotated) datasets. Furthermore, as the values reported for the two drug variables used in QEUH are for continuous delivery and not for occasional boluses, it was important to determine that the drug delivery modes are equivalent.
iii. The QEUH datasets report information on an hourly basis, whereas the reporting of data in the external datasets is both more frequent and at irregular intervals, so considerable effort was expended to transform both the HiRID & MIMIC-III datasets to “hourly” datasets, so these datasets would be compatible with the classifiers derived for the QEUH consultants. See the ‘Code Availability’ section for details on accessing the complete HiRID pre-processing steps.

### Inter-annotator agreement metrics

Inter-annotator agreement (IAA), also called inter-rater reliability, is a measure of the extent to which the annotators assign the same category to the same instance. IAA represents the consistency of annotations, as well as the reproducibility of the labelling task. High consistency is favoured as this minimises errors due to subjectivity and increases reliability in the training data.

There are multiple statistics used to measure IAA, including Cohen’s *κ*, Fleiss’ *κ* and Krippendorff’s *α*. All three statistics were calculated within Python 3.0 using: cohen_kappa_score from sklearn.metrics^60^, fleiss_kappa from statsmodels.stats.inter_rater^61^, simpledorff^62^.

Cohen’s *κ* measures the reliability between two annotators, considering the possibility of the agreement occurring by chance. Cohen’s scale can be summarized as: 0.0–0.20 (None); 0.21–0.39 (Minimal); 0.40–0.59 (Weak); 0.60–0.79 (Moderate); 0.80–0.90 (Strong); > 0.90 (Almost Perfect)^32^.

Fleiss’ *κ* is an extension of Cohen’s κ which considers the consistency of annotator agreements, as opposed to absolute agreements. It assesses the reliability of agreement across multiple annotators. Fleiss’ scale can be summarized as: < 0 (Poor); 0.0–0.20 (Slight); 0.21–0.40 (Fair); 0.41–0.60 (Moderate); 0.61–0.80 (Substantial); 0.81–1.0 (Almost perfect)^34^.

Krippendorff’s *α*^63^ considers the consistency of annotator agreements, as opposed to absolute agreements. It assesses the reliability of agreement across multiple annotators.

### Patient population compatibility

Systematic reviews on model validation studies have shown a lack of well-conducted and clearly reported external validation studies^55,56^. A detailed investigation of the compatibility between the training and validation datasets, including patient populations, is uncommon, yet necessary to improve the reliability of external validation.

Within this study, to assess the patient population compatibility between the training and validation datasets, Adrenaline/Noradrenaline administration was investigated. Adrenaline/Noradrenaline is administered to patients whose cardiovascular system is unstable and indicates a high severity patient status. Only 5.9% of the MIMIC-III ICU admissions were administered Adrenaline/Noradrenaline, compared to 31.5% of the HiRID ICU admissions. This indicates the severity of ICU patients in the Bern University Hospital, Switzerland, was higher than in the Beth Israel Deaconess Medical Centre, US. Furthermore, 40% of the QEUH ICU training instances were administered Adrenaline/Noradrenaline. This indicates the ICU patient population within the training data has higher severity conditions and therefore has good compatibility with HiRID, whereas poor compatibility with MIMIC-III. So, we decided to use HiRID as the validation dataset in this study. (Note, because we are undertaking a study to predict whether patients are discharged alive or die in the ICU, it is important to have a significant number of both these events in the validation dataset).

### External validation experiment 1: Preparation of the validation dataset

This experiment tests the classifiers’ ability to classify patient discharge outcomes (alive or dead), under the assumption that the patient’s physiological/pharmacological status within the last hour before discharge/death is a good indicator of their discharge status. The “full” HiRID dataset which resulted from the pre-processing discussed above has 2,022,313 instances sourced from 20,073 unique ICU admissions. Only time-points which are recorded in the dataset as corresponding to discharged alive or dead within the next hour, were eligible for selection. 1300 “Discharged Alive from ICU” and 1300 “Died in ICU” instances were randomly selected as the validation dataset.

After discussion with ICU professionals, we established ‘discharged alive from ICU’ usually indicates the patient is discharged from ICU to a non-ICU hospital ward (rather than discharged from the hospital). Data around discharge location or readmission to ICU was not provided in the HiRID database. In our study, discharge location does not impact our experimental approach or findings, as the “Discharged Alive from ICU within 1 h’' cohort still represents the most stable patients (i.e., ICU-PSS = A).

### External validation experiment 2: Investigating time-series validation datasets

In reality, ICU consultants consider the trend in patient physiological and pharmacological parameters across the period of time before making their assessment. To capture this real-world ICU patient severity classification task more closely, we ran a second external validation experiment on HiRID time series data and compared the performance of the 11 DT classifiers (trained on the QEUH annotated datasets) on static and temporal HiRID validation datasets. All validation datasets contain the same 6 variables as in the training dataset (Adrenaline, Noradrenaline, FiO_2_, SpO_2_, MAP, Heart Rate).

To assess performance of the classifiers on the HiRID temporal validation datasets, the weighted sum of the five (hourly) ICU-PSS predictions per patient. The hourly weights were defined as follows, giving more weighting to the readings closer to discharge/death: (a) 5 h before discharge/death: 0.1, (b) 4 h before discharge/death: 0.1, (c) 3 h before discharge/death 0.2, (d) 2 h before discharge/death: 0.3, (e) 1 h before discharge/death: 0.3. Note, time periods longer than 5 h were investigated for use in this experiment, however these resulted in smaller validation datasets – a period of 5 h provided an optimal balance between enough time series datapoints per patient and validation dataset size.

The A-E predicted labels were treated as a 1–5 ordinal scale, therefore the weighted sum values were all in the range 1–5. The trained models were treated as predicting three classes: CL1 = A, CL2 = B/C/D, & CL3 = E.

In the Results section, two methods of mapping the weighted sum values (1–5) to these three classes were reported, with differing cut-offs:

i. ‘Extreme’: CL1 = 1, CL2 = > 1–4, CL3 = > 4.
ii. ‘Neutral’: CL1 = ≤ 3, CL2 = > 3-<4, CL3 = ≥ 4.

We also investigated an additional ‘Extreme (2)’ cut-off with weighted sum mapping shown below. These results are outlined in Supplementary Fig. 1.

iii. ‘Extreme (2)’: CL1 = ≤ 2, CL2 = > 2-<4, CL3 = ≥ 4.

In further analysis, DT and LR models were trained on the predicted labels made by the 11 QEUH DT classifiers on the temporal HiRID validation dataset, for each of the five hours before discharge/death (i.e., combining ICU-PSS labels across five consecutive hours). This is a simple but interpretable approach to mimic the ICU doctors’ decision-making process, which consider patterns of change across patient pharmacological/physiological parameters, before making a discharge decision. More complex models with non-linear kernels, such as SVM, may be used for this analysis – however this would lose the interpretability of the results, The DT and LR models were optimised on F1 micro and evaluated via 5-fold cross validation, where the dependent variable is actual discharge status (see Supplementary Fig. 2).

**Fig. 9:**
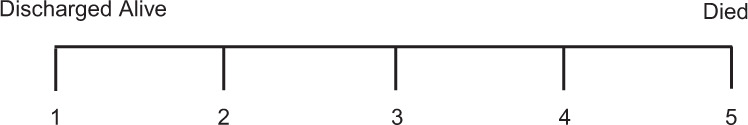
The ‘fuzzy’ prediction scale. Specifically, the predicted labels 1–5 (i.e., A–E) on an ordinal scale where the two extremes represent the binary classification task: 1 = Discharged alive from ICU within the next hour, 5 = Died in ICU within the following hour.

Within this second external validation experiment, in addition to the MV and TMV consensus models, an additional ‘Fuzzy Consensus’ (FC) model was built. This purpose of this building FC model is to investigate combining the individual models’ outputs by considering their outputs as confidence values for binary classification task on the external validation dataset (discharged vs death). In this consensus method the all predictions are captured and interpreted as ‘fuzzy’ labels, on an ordinal scale of 1–5 (i.e., A-E), when calculating the overall discharge status prediction for each patient. Figure 9 illustrates the scale used.

## Supplementary information

Supplementary Material

## Data Availability

The QEUH training data that support the findings of this study may be available on request from the data controller and co-author, Malcolm Sim. The data are not publicly available as individual level healthcare data are protected by privacy laws. The HiRID and MIMIC-III are publicly accessible at the following URLs: 1. MIMIC-III database: https://mimic.mit.edu/docs/gettingstarted/. 2. HiRID database: https://www.physionet.org/content/hirid/1.1.1/.

## Code availability

For reproducibility, all dataset pre-processing and machine learning model codes for this study is accessible here: https://github.com/aneeta-sylo/npjDigitalMedicine. The external validation datasets and machine learning models were constructed using Python 3.6.
