# Supplementary material for: The impact of inconsistent human annotations on AI driven clinical decision making
Source: NPJ Digit Med. 2023 Feb 21;6:26. doi: 10.1038/s41746-023-00773-3 (PMC9944930; doi:10.1038/s41746-023-00773-3)
Supplement: Supplementary file 1 — Supplementary Material [file 41746_2023_773_MOESM1_ESM.pdf]

Supplementary Tables & Figures

Class label	Description
A	Patient's cardiovascular system (CVS) normal, with no adrenaline or noradrenaline and low levels of oxygen; urine production often essentially normal (or is well established on renal replacement therapy).
B	Patient CVS nearly normal, probably needs low levels of adrenaline or noradrenaline and oxygen.
C	Patient CVS system is effectively stable; probably on moderate dosages of adrenaline or noradrenaline and oxygen.<br>Most parameters suggest the time-slot is in category A or B, but if any of the following conditions are met, then it should be assigned to category C:<br>- heart rate: moderately low or moderately high;<br>- MAP: moderately low or moderately high;<br>- adrenaline: moderate dose;<br>- noradrenaline: moderate dose;<br>- FiO2: moderate;<br>- SpO2: moderately low.
D	Patient's CVS system is moderately unstable and/or on high doses of adrenaline or noradrenaline or fluid to retain stability.<br>Most parameters suggest the time-slot is in category A or B, but if any of the following conditions are met, then it should be assigned to category D:<br>- heart rate: low or high;<br>- MAP: low or high;<br>- adrenaline: high dose;<br>- noradrenaline: high dose;<br>- FiO2: high;<br>- SpO2: low.
E	Patient's CVS is very unstable (which is usually true in early phases of resuscitation or following a new event) with low BP and high HR or rapidly changing adrenaline or noradrenaline dosage and requires substantial fluid inputs.<br>Most parameters suggest the time-slot is in category A or B, but if any of the following conditions are met, then it should be assigned to category E:<br>- heart rate: extremely low or extremely high;<br>- MAP: extremely low or extremely high;<br>- adrenaline: extremely high dose;<br>- noradrenaline: extremely high dose;<br>- FiO2: extremely high;<br>- SpO2: extremely low.

Supplementary Table 1. High-level description of the ICU-PSS scale.

Annotator	Internal Val. F1 micro	External Val. F1 micro
C1	0.600	0.262
C2	0.750	0.541
C3	0.650	0.328
C4	0.700	0.425
C5	0.583	0.230
C6	0.517	0.175
C7	0.550	0.358
C8	0.800	0.423
C9	0.583	0.430
C10	0.650	0.168
C11	0.567	0.302

Supplementary Table 2. Internal and external validation performance of the Random Forest models across all 11 consultants (C1-C11), using balanced class weights.

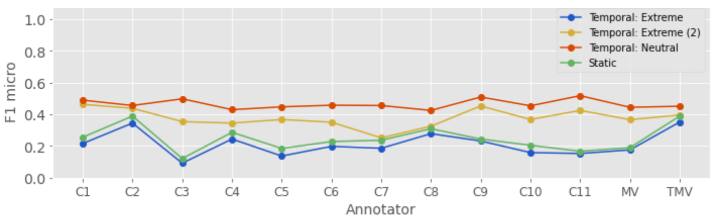

Supplementary Figure 1. Comparison of the RF classifiers' external validation performance on static vs temporal HiRID validation datasets.

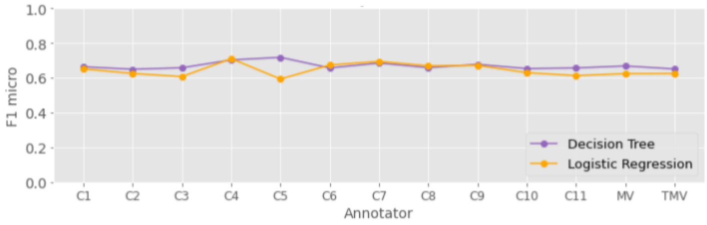

Supplementary Figure 2. Internal validation performance of the supervised DT and LR models trained on temporal HiRID dataset with predicted labels.

a

Annotator	Internal Val. F1 micro	External Val. F1 micro
C1	0.62	0.13
C2	0.67	0.51
C3	0.57	0.24
C4	0.63	0.40
C5	0.57	0.22
C6	0.53	0.21
C7	0.57	0.28
C8	0.78	0.41
C9	0.50	0.27
C10	0.57	0.15
C11	0.57	0.15
MV	0.63	0.31
TMV	0.65	0.44

b

Annotator	Internal Val. F1 micro	External Val. F1 micro
C1	0.38	0.45
C2	0.67	0.57
C3	0.55	0.34
C4	0.55	0.34
C5	0.38	0.23
C6	0.50	0.20
C7	0.45	0.43
C8	0.68	0.41
C9	0.52	0.32
C10	0.53	0.16
C11	0.55	0.34
MV	0.62	0.37
TMV	0.55	0.32

Supplementary Figure 3. (a) Internal & external validation performance of the supervised XGBoost models, trained on the QEUH datasets. (b) Internal & external validation performance of the supervised SVM models, trained on the QEUH datasets.
